# Dysmenorrhœa

**Published:** 1910-05-14

**Authors:** R. A. Gibbons

**Affiliations:** Physician to the Grosvenor Hospital for Women.


### Hospital Clinics.

### DYSMENORRHŒA (*continued*).

By R. A. GIBBONS, M.D., F.R.C.S.; Physician to the Grosvenor Hospital for Women.

(A Lecture delivered at the Medical Graduates' College and Polyclinic, January 26, 1910.)

And now we come to the treatment of dysmenorrhœa, which is, after all, the most important from a practical point of view. For although theories as to pathology and ætiology are most interesting to study, they do not advance us at all if they do not help us to relieve pain and to cure disease.

### A Note on Treatment.

Treatment may be easily divided into therapeutic and operative. Before passing to either it may be well to say a few words about prophylaxy. I have seen enough to know that there are many girls suffering from severe dysmenorrhœa who have come under my observation, having started the period in an ordinary painless manner, who have been quite healthy, and who have developed dysmenorrhœa apparently from causes which might have been averted. I could give many cases bearing on this, but the time at our disposal is limited, and I shall only mention one in illustration of what I mean. A young lady twenty-three years of age was brought to me on account of dysmenorrhœa, dating from about a year before. She commenced her period at thirteen, and was always regular, suffering no pain, and never being troubled in any way by it. She was staying at a country house in Scotland, and on the first morning of her period started a drive of four hours in an open dog-cart. Rain fell, and continued heavily during her drive; she was soon cold and wet, and told me that for more than two hours she was sitting in a pool of water. Pain came on within a few hours, and this attack was followed by regular severe dysmenorrhœa. This case alone shows that dysmenorrhœa may be induced, and I have had many similar severe cases brought on by cold travelling. It is only prudent to advise girls to be careful of themselves, without making any fuss, at the time of the period. I see no reason to advise a healthy girl to remain in bed for the first day because of her period coming on; but I know that many brought up in luxury are made by their mothers to do so. If a girl is really healthy, beyond giving directions for sensible care at the time of the period nothing more need be done. It is a mistake to educate healthy girls to the belief that it is necessary to look upon the period as an illness. There are, unfortunately, only too many who are really delicate, and who are obliged to look upon themselves as invalids for so many days of every month; but for those who do not suffer, and who enjoy good health, they may go about their lives as usual, merely taking care to avoid any violent exercise. It is also important to avoid, if possible, passive chill, and for girls to remember to clothe warmly during cold weather.

The other cause which I believe will induce dysmenorrhœa in those who have not previously suffered from it is over-fatigue, often most difficult to avoid; but cold and over-fatigue together are, in my opinion, a fertile cause of the onset of dysmenorrhœa. If a girl who has a good family history, who has health to start with, lives an ordinary healthy life, with plenty of fresh air, attending to hygienic laws, she apparently ought not to become subject to dysmenorrhœa. About this, however, strangely enough nothing positive can be said, for sometimes the most healthy-looking girls gradually develop dysmenorrhœa, and others who are delicate, anæmic, and feeble pass through each period without any trouble. It is impossible to forecast the future menstrual history of any girl who is commencing womanhood, however well she may be at the onset. I have very frequently observed that there is a family history of dysmenorrhœa for several generations. In ordinary slight cases of this disease, where a girl is complaining of malaise and headache, with only a little pain, rest and warmth will probably be sufficient. The application of dry heat in the form of hot-water bottles and simple hot drink will probably be all that is required for relief. As to the drink, it may be hot barley-water, milk and water, or the old-fashioned mulled wine, or hot spirit and water. In the slight cases hot fluid is sufficient, whatever it may be, and the addition of alcohol is unnecessary, especially if the patient is not in the habit of taking any. If the pain is not rapidly overcome by this, then some ordinary analgesic medicine may be given, of which I shall speak directly.

### Rest in Bed.

In the severe cases the patient does not need to be asked to go to bed, for, as a rule, she must be there. In some cases I have already referred to, where, although the pain is very severe, it is not sufficient to cause sickness or faintness, the patient may be able to keep about. This entirely depends upon the nervous system of the individual and the power of self-control. As a rule it is impossible to keep about during the whole time, and retirement for even an hour or two is necessary. In such cases the pain may be overcome by immediate treatment. As soon as the first indication of pain occurs, if it be by day, and it is possible to do so, a hot Sitz bath of ten or fifteen minutes' duration is often of the greatest relief. An ordinary hot bath is of no use; the patient must immerse the middle of the body in the bath, the legs and upper part of the body being still covered. It is best to add hot water as the water cools, so that the temperature is kept up, and the patient may have it as hot as she can comfortably bear it. After this, if she is put into a well-warmed bed, with an indiarubber hot-water bottle to the back, and one on the abdomen, great comfort will be given. It is of importance to let the patient lie on a hot-water bottle, because there is usually so much sacral pain, and the dry heat affords immense relief. Sometimes the abdomen is so tender that the hot-water bottles cannot be borne.

In these cases I advise a very thick layer of wool, which has previously been baked in the oven until quite hot (this can be done in an ordinary tin box), to be applied all over the abdomen, sometimes sprinkled with laudanum. It is light, and can be easily borne. If the abdomen is very tender, the application of equal parts of liniment of aconite and liniment of belladonna is particularly soothing, combined with the heat. At the same time I order a teaspoonful in a wineglassful of water of granular effervescing phenacetin, containing either five grains or ten grains to the drachm, according to the severity of the pain. If not better in twenty minutes, this is to be repeated with one drachm of brandy, and if in another twenty minutes relief does not come, I give a third dose, with another drachm of brandy. The pain is usually relieved after two or three doses. It is best to give a little alcohol with the phenacetin, to prevent the depressing effects on the heart. Should it be necessary, a dose can be given every four hours after the second or third doses have been given.

### The Relief of Pain.

In a certain number of cases vomiting comes on early with the pain, and whatever is ordered may be rejected. It is usually better to order the effervescing mixture wherever there is a tendency to nausea or sickness, as it seems to be more easily borne, but some may prefer it in the form of cachet or powder. Should vomiting be frequent, mustard to the epigastrium may be applied, often with good results. It is well to bear in mind that when the pain is soothed and vomiting has ceased the patient should not be allowed to get up, or make any effort too soon, or the pain and nausea or sickness may recur immediately on exertion. Should no vomiting or nausea be present, nourishment in some easily digested form should be given during the period of pain. Sometimes, if the pain comes on before the flow, much relief may be obtained by a pint of hot saline solution given per rectum with thirty or forty grains of bromide of sodium or ammonium added, which must be retained to be of service. There are certain cases where this seems to answer well, but if relief can be obtained by other means I prefer them. A hot saline injection with a fair dose of phenacetin may be given if the patient is too sick to take anything by the mouth. Fifteen grains may be given, and repeated later should it be necessary, but brandy ought to be given at the same time mixed with the injection, to counteract the depressing effects of the phenacetin.

The old-fashioned foot-bath, with a little mustard, is often invaluable in certain cases. Emmett recommended in addition a hot mustard plaster about three inches in width, reaching from the cervical region of the spine to the sacrum,[18] and also dry cupping when the flow is delayed, which I have seen give great relief.

### Some Useful Drugs.

For the agonising form, if large doses of phenacetin or such-like drugs do not afford relief, the only remedy is morphia hypodermically. Fortunately these cases are rare, and recourse to large doses of morphia is unnecessary; but it is well to always remember the danger of giving morphia to a patient suffering from dysmenorrhœa, for the habit of taking morphia is easily established, and as the pain recurs every month the temptation to increase the dose is ever present. In giving it for these most severe cases, it is better to combine it with atropine, to avoid the risk of sickness. For phenacetin may be substituted aspirin, antipyrin, antifebrin, novaspirin, castor, chloretone, or any similar drug, and occasionally nitrite of amyl, or nitro-glycerine; but it must be remembered that all these drugs only soothe the pain and tide over the attack. What we have to study is the cure of the disease, and none of the drugs I have named will do this. Unfortunately this disease has no tendency to become cured without treatment. We must endeavour to find a drug which will so act as to prevent the onset of this severe pain, or at any rate greatly mitigate it, so that when the period occurs the patient will not suffer much. Now, one of the drugs which is most commonly used is guaiacum. The late Dr. Matthews Duncan, with whom I often discussed this affection, placed it before everything, and Dr. Herman speaks well of it. It is, no doubt, often excellent, and it is best given at least a week before the period is due, in doses of ten grains three times a day. I have not found it of as much service as tannate of cannabin, which, in my experience, seems to have done more good than anything I have tried. I give it in the form of a pill three times a day after meals, for seven days before the period is expected. It may be given in doses of from two to three or four grains, and if preferred may be continued through the period in increased doses. I usually stop it when the pain comes on if at all bad, and give one of the coal tar analgesic remedies; but if the pain is not severe, the tannate of cannabin may be continued until all chance of recurrence of the pain has disappeared. With it I frequently give ovarian substance. The treatment ought to be continued for some months before discarding it, for it is well to persevere with it. The only disagreeable symptom I have known, and that has been quite rare, has been a certain amount of looseness of the bowels. Of course, if there is any indication of this the drug must be stopped. This is the only point worth mentioning about its administration. I used Cannabis Indica for a considerable time, and although it was often of great service, I frequently found that it produced a mild form of delirium or excitement, and as this was sometimes alarming to the friends of the patient I gave it up, and now never use it. Bromides are often of great service, but they ought to be commenced some time before the onset of the period, and are apt to cause in some patients a certain amount of depression. Amongst other drugs, I have often found Viburnum Prunifolium of service, as well as the salicylate of soda. It would be useless to enumerate all the drugs which have been advocated in the treatment of this disease. I have had better results from the use of cannabin tannate than from any other drug. I believe it acts, as all drugs which benefit do, by soothing the hyperæsthetic mucous membrane.

I must merely mention the treatment by Fleiss, of the application of cocaine to the "sexual spots" on the nasal mucous membrane. In a paper by Dr. Koblanck[19] on nasal reflexes, he advocates the application of cocaine to the so-called sexual spots in the nasal mucous membrane in some forms of dysmenorrhœa. I need not go further into this treatment, for I have had no experience of its use. Kolischer[20] attributes good results to suggestion. In many cases I have certainly done good by the administration of ovarian substance, and I have found that when used with girls who appeared anæmic it answered well when combined with iron or arsenic. It may be that my theory of the disease explains its action. The administration of ovarian substance must be watched, for sometimes it causes nausea. I usually give tablets of five grains (containing 0.32 gra.) two or three times a day after food.

Before passing to operative measures I must refer to Boult's,[21] who considers that there is an antagonism between the physiological function of the ovary and mammary gland, and that menstruation is to be conceived of as a process which is set going by the biological power of the ovary. He has attempted to overcome the intensity of the pathological conditions of menstruation, and, above all, of dysmenorrhœa, by artificial stimulation of the mammary gland. He has employed Klapp's breast pump, and has produced, some days before menstruation, and during it, engorgement of the mammary gland. He states that he has seen good results follow this treatment, both in the abatement of the pain and in the diminution of the flow of blood.

### The Question of Operation.

And, lastly, we come to the surgical, or operative treatment. This may be briefly divided into dilatation of the cervix, and removal of the ovaries, the latter not to be undertaken without the most serious consideration. Now dilatation is, on the whole, a most successful operation, and all those who have had special experience in the treatment of this disease are favourably impressed by the results. It imitates nature's cure, which is pregnancy, for then thorough dilatation of the cervix is secured, but unfortunately we know that dysmenorrhœa is frequently associated with sterility, so that this natural cure is often denied the patient. Some prefer to incise the os laterally before dilating, or to cut out a wedge-shaped piece from the cervix all round the canal.[22] Others divide the cervix, stitching back the angles, so as to insure their remaining permanently patent. In my own practice I hardly ever incise the cervix. It need only be done at the time of dilating if the os is extremely small. Incision of the internal os, which involves unnecessary risk, is practically never done now. It used to be much in vogue, and gave relief probably by dividing the circular fibres and giving them a rest from spasm. This object is, of course, gained by slow dilatation of the cervix. As a rule, I use the ordinary Hegar's metal dilators, and do the operation at once under an anæsthetic. If the patient has not the time to devote to rest afterwards it is better not to do it at all.

### Dilatation.

The operation of dilatation should be accompanied with the same precautions as for any other surgical operation, and it is well to bear in mind that there ought to be no such thing as a minor operation in surgery, because every operation undertaken ought to be carried out with scrupulous care. After thorough dilatation it is best to keep the patient warm in bed for a few days. Some advocate the use of electricity in the treatment of dysmenorrhœa,[23] or the intra-uterine application of the galvanic current,[24] and by others the stem pessary is lauded. I have used neither, and I do not advise the use of the stem pessary, for it cannot be altogether free from risk. It may be that I am prejudiced against it on account of the unfortunate results I have seen from its use. In cases of dysmenorrhœa where there is continued pain over one or both ovarian regions, it is found sometimes on opening the abdomen—the operation being possibly undertaken for something else—that the ovaries may have small cysts all over them. If these cysts be punctured, and the fluid allowed to run out, the ovarian pain will sometimes disappear, because, presumably, the tension is relieved. When intense pain over one ovary or both is invariably complained of at the onset of or during each menstruation, pain sufficient to incapacitate and prevent the patient from making her living, it may be justifiable to explore. In certain cases the ovary may be found to be cystic, as above mentioned, and in some cases I have seen there have been adhesions from previous local peritonitis. If these are thoroughly broken down without doing anything further, the patient may be cured.

### Gradual Dilatation.

I believe some do gradual dilatation: that is, passing a dilator increasing in size every two or three days until the full size is reached. I do not recommend this proceeding. It is tedious and somewhat risky, and certainly very painful. I have known parametritis to follow in the practice of more than one who passed the dilators in his own house. It is best to operate under an anæsthetic, and to pass each dilator slowly, so that the cervix may be slowly and steadily dilated and not torn. My own experience is that if the dilators pass with considerable difficulty there is a much better prospect of a cure than if they enter the cervix easily.

And now, finally, there is oophorectomy. This is a matter upon which the patient, and she alone, ought to be the judge. It must, of course, be most strongly condemned unless done only after the most serious consideration. If the woman is of a certain age, shattered in health, with no prospect of relief in spite of all kinds of treatment, and if she is willing to relinquish the chance of child-bearing, then I think that her request for the operation, which will stop menstruation and cure her pain, ought to be attended to. I would say this, however, that her statements as to her sufferings ought to be corroborated by the relations or friends with whom she lives. No one would undertake this operation without feeling satisfied that the patient had had everything for and against placed before her, and, above all, it is essential that absolute certainty should prevail as to the diagnosis of the disease. I have already referred to dysmenorrhœa and the cure of neurasthenic patients. Be certain, before undertaking an operation of this kind, that the patient is really suffering from genuine spasmodic dysmenorrhœa, and that she is not a neurotic woman, with pain intensified at the time of menstruation.

In conclusion, I would say with much regret that I do not consider we know any more about dysmenorrhœa which will enable us to speak positively as to its ætiology or pathology than we did years ago, but I cannot help feeling that further observations concerning the secretions of the ovary may open up a wide field of knowledge. The exact relation between the ovary, the thyroid, and the other ductless glands is still obscure,[25] but we must earnestly hope that the time is not far distant when we shall be enabled to speak positively about the action of their important secretions, and that a true explanation of the occurrence of dysmenorrhœa may be forthcoming.

[18] *Principles and Practice of Gynæcology*, 3rd vol. 1884, p. 177.

[19] *Zentralblatt für Gynækologie* 1908. XXII., p. 581, Gesellschaft für Geburtschilfe und Gynækologie in Berlin.

[20] *Amer. Journal Obst.* Vol. 49, p. 804.

[21] Zur Behandlung der Dysmenorrhœa. *Münchener Med. Wochenscrift*, 1907, 44, 1731.

[22] A Type of Operative Dysmenorrhœa, by Gustav Kolischer. *Amer. Jour. of Obstetrics*, 1909, 59, 644.

[23] W. B. Sprague, *Amer. Gyn. and Ped.* 1891, Vol. 4, p. 402.

[24] A. Lapthorn Smith, *Amer. Jour. Obst.* 1896, Vol. 26, p. 161.

[25] Dr. Leonard Williams, *Chir. Jour.*, p. 327, March 3, 1909. Dr. T. G. Stevens, *Chir. Jour.*, August 11, 1909.

